# Unilateral palpebral lupus miliaris disseminatus faciei successfully treated with lymecycline^؆^

**DOI:** 10.1016/j.abd.2025.501219

**Published:** 2025-11-06

**Authors:** Soledad Aspillaga, Jaime Perez-Wilson, Alex Castro, Edinson López, Fernando Lohse

**Affiliations:** a Department of Dermatology, Clínica Alemana de Santiago, Universidad del Desarrollo, Santiago, Chile; b Department of Pathology, Clínica Alemana de Santiago, Santiago, Chile

*Dear Editor,*

A 26-year-old male patient with no previous relevant medical history presented with small reddish and yellowish papules on the right upper eyelid, which had evolved over 2 months. These lesions were asymptomatic. On physical examination multiple micro-papules, 1 to 2 mm in diameter, grouped on an erythematous base and confluent on the right upper eyelid. Dermoscopy showed rounded, polylobulated lesions which spared follicular openings, with an orange-reddish background and short telangiectatic vascular structures surrounding the follicular openings (Fig. 1).

**Fig. 1:**
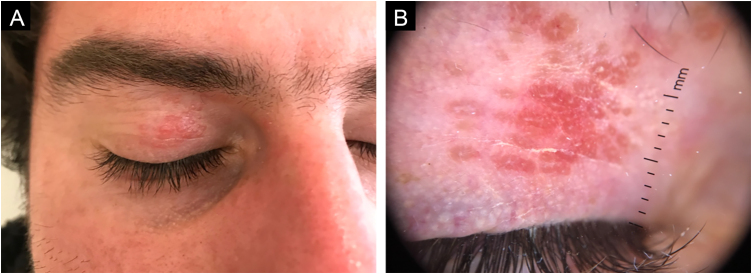
(A) Multiple micropapules, 1 to 2 mm in diameter, grouped on an erythematous base and confluent on the right upper eyelid. (B) Dermoscopy shows polylobulated lesions with a rounded appearance, sparing follicular openings, on an orange-reddish background, and short telangiectatic vascular structures surrounding follicular openings.

Initially, the patient was treated with topical fusidic acid under the suspicion of folliculitis of the eyelid. However, after 2-weeks without improvement, a punch biopsy of a papular lesion was performed. Histological findings revealed orthokeratosis with preserved epidermal structure. In the dermis, an area of necrosis surrounded by granulomas composed of numerous epithelioid histiocytes, a few giant cells, and lymphocytes was found, consistent with the diagnosis of lupus miliaris disseminatus faciei (LMDF) (Fig. 2).

**Fig. 2:**
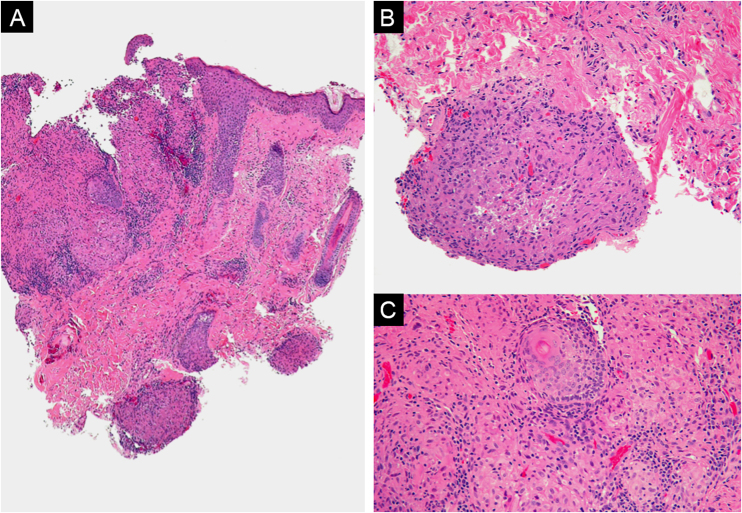
(A) Multinodular dermal inflammatory infiltrate (Hemaxitolyn & eosin, ×40). (B) The infiltrate consists of lymphocytes, plasma cells, and histiocytes forming epithelioid granulomas (Hemaxitolyn & eosin, ×200). (C) Some granulomas show central necrosis (Hemaxitolyn & eosin, ×200).

Based on clinical and histopathological findings, a diagnosis of LMDF was established. Treatment with oral lymecycline 300 mg/day and topical metronidazole 0.75% twice a day for 2-months was prescribed. At the end of treatment, complete regression of the lesions was observed, which has been maintained after 16 months of follow-up.

Lupus miliaris disseminatus faciei or acne agminata is a chronic inflammatory granulomatous disease affecting adolescents and young adults, with a male predominance.^1^ Clinically, it manifests as multiple erythematous-yellowish or brown papules, 1‒3 mm in diameter, involving the central face and eyelids, especially the lower eyelids, although they are rarely the only site of involvement. Additionally, this condition is usually bilateral, unlike our case, where the involvement was unilateral and on the upper eyelid. Typically, the lesions resolve spontaneously after 1 to 2 years, leaving small punctate scars on the skin.^1^

On diascopy, the papules often have an “apple jelly” appearance.^2^ Dermoscopy displayed the presence of central targetoid follicular plugs with white striae on a brownish-orange background. The keratotic follicular plugs seen in dermoscopy develop as a result of lateral pressure on the hair follicles.^3^

In the past, LMDF was considered a variant of lupus vulgaris or a tuberculoma, due to histopathological features showing epithelioid cell granulomas with central caseous necrosis. However, in these patients, methods to demonstrate the presence of tuberculous mycobacteria are consistently negative.^1^ Some authors have considered LMDF synonymous with granulomatous rosacea due to similarities in histopathological characteristics. However, both conditions have clinical and histopathological differences and respond to different treatments.^4^ Currently, it is believed that the condition occurs secondary to the rupture of the hair follicle, releasing antigens ‒ primarily from *Cutibacterium* acnes ‒ into the dermis, triggering a foreign body reaction via cellular immunity. This theory is based on pathological observation showing granulomas forming around ruptured pilosebaceous follicles.^5^

Definitive diagnosis requires histopathology, which typically shows perifollicular epithelioid granulomas, neutrophilic abscesses, and caseous necrosis in 13%‒48% of cases.^5^

The differential diagnosis of LMDF includes granulomatous rosacea (GR), lupus vulgaris, papular sarcoidosis, the papular form of granuloma annulare, and chalazion.

The most important differential diagnosis is granulomatous rosacea.^4^ Both conditions show epithelioid granulomas centered on pilosebaceous units in histopathological studies; however, the granulomas of GR are smaller and lack central necrosis. Clinically, GR can be distinguished by presenting flushing, erythema, and centrofacial telangiectasias, unlike LMDF, which only presents papules.^4^

Since the etiology and pathogenesis remain unclear, there are no standard treatment recommendations. However, lesions resolve spontaneously, although they leave small, depressed scars. Case series have reported a wide range of treatments aimed at faster lesion resolution and scar prevention.^6^ These include dapsone, doxycycline, minocycline, isotretinoin, clofazimine, isoniazid, intralesional triamcinolone, oral corticosteroids, 1450 nm diode laser, and, more recently, apremilast.^6^

In 2020, Kaushik et al. reported the use of apremilast 30 mg every 12 hours in a series of 3 patients who were refractory to other treatments such as doxycycline and isotretinoin. In this series, the 3 patients showed rapid improvement within 4 weeks without adverse effects.^6^

## ORCID IDs

Soledad Aspillaga: 0009-0009-6840-0565; Jaime Perez-Wilson: 0009-0008-5229-8319; Alex Castro: 0000-0003-4431-5293; Edinson López: 0000-0001-7531-7836; Fernando Lohse: 0009-0002-1076-6723

## Authors' contributions

Soledad Aspillaga: The study concept and design; data collection, or analysis and interpretation of data; writing of the manuscript or critical review of important intellectual content; critical review of the literature; final approval of the final version of the manuscript.

Jaime Pérez-Wilson: The study concept and design; data collection, or analysis and interpretation of data; writing of the manuscript or critical review of important intellectual content; critical review of the literature; final approval of the final version of the manuscript.

Alex Castro: The study concept and design; data collection, or analysis and interpretation of data; writing of the manuscript or critical review of important intellectual content; critical review of the literature; final approval of the final version of the manuscript.

Edinson Lopez: The study concept and design; data collection, or analysis and interpretation of data; writing of the manuscript or critical review of important intellectual content; critical review of the literature; final approval of the final version of the manuscript.

Fernando Lohse: The study concept and design; data collection, or analysis and interpretation of data; writing of the manuscript or critical review of important intellectual content; critical review of the literature; final approval of the final version of the manuscript.

## Financial support

None declared.

## Research data availability

Does not apply.

## Conflicts of interest

None declared.

## References

[1] Chougule A., Chatterjee D., Yadav R., Sethi S., De D., Saikia U.N. (2018). Granulomatous rosacea versus lupus miliaris disseminatus faciei-2 faces of facial granulomatous disorder: a clinicohistological and molecular study. Am J Dermatopathol.

[2] Alonso V., Ramón D., Martín J.M., Monteagudo C., Molina I., Jordá E. (2005). Lupus miliaris disseminatus faciei. Actas Dermosifiliogr.

[3] Ayhan E., Alabalik U., Avci Y. (2014). Dermoscopic evaluation of two patients with lupus miliaris disseminatus faciei. Clin Exp Dermatol.

[4] Lee G.L., Zirwas M.J. (2015). Granulomatous rosacea and periorificial dermatitis: controversies and review of management and treatment. Dermatol Clin.

[5] Liao W., Jolly S.S., Brownstein S., Jordan D.R., Gilberg S., Prokopetz R. (2010). Lupus miliaris disseminatus faciei of the eyelids: report of two cases. Ophthal Plast Reconstr Surg.

[6] Kaushik A., Kumaran M.S., Chatterjee D., De D. (2020). The search for a uniformly effective treatment in patients with lupus miliaris disseminatus faciei. JAMA Dermatol.

